# The After-Histories of Sanatorium Patients

**Published:** 1914-09-19

**Authors:** 


### THE AFTER-HISTORIES OF SANATORIUM PATIENTS.

### A Report from Frimley. By a Tuberculosis Officer.

A report which deserves the attention of all tuberculosis officers, sanatorium physicians, and medical officers of health, is the one which has been issued from the Brompton Hospital Sanatorium at Frimley. It is but brief as regards text yet is very rich indeed in statistical tables. Of these there are thirty-six, showing the figures relating to the condition of certain sets of cases at the end of one to five years after discharge. Before considering these tables it must be borne in mind that the results obtained at Frimley cannot be taken as giving any accurate index of the results which might be expected if all cases of tuberculosis of the lung were sent in a haphazard way to sanatoria.

### The Class of Case.

It is well known that of the patients treated at Frimley the great majority have first been in the wards of the Brompton Hospital. Those sent to the sanatorium were carefully selected, not only from the clinical point of view, but also, and this is an important point, from the economic aspect: preference was given to those who had a certain or reasonable chance of obtaining work after their discharge. While at Frimley the patients pass through the courses of exercise and work which were ably adapted by Dr. Marcus Paterson. These details are to be found fully set out in the report and need not detain us now.

The actual cases under consideration are those who were discharged during the years 1905-1910. These amounted to 1,892, but 218 of these are deducted for adequate reasons, and the remaining 1,674, comprising 1,183 males and 491 females, are classified in the tables in four divisions according to their condition during each year following their discharge. These divisions are:—(1) Well and able to work; (2) Alive; (3) Known to be dead; (4) Lost sight of.

It should be noted that those patients who describe themselves as being able "occasionally to do a little light work," have been relegated to class 2. The numbers in class 4 are somewhat large—a difficulty with which all sanatoria have to contend. The classification of the extent of disease also comprises four divisions, according to the number of lobes of the lung in which physical signs were found, but those cases with indefinite or no physical signs have been classed with those in the first group in whom signs were present in one lobe. The second and third groups include those cases with signs in two and three lobes respectively; while the fourth group includes those with disease in four or five lobes. A praiseworthy duplication of the tables was necessitated by the decision to tabulate separately the patients in each year in whose sputum acid-fast bacilli were found, and to provide distinct sets of figures for those in whose sputum the bacilli were not found. The first class number 1,176, the second 498, and were diagnosed on other grounds.

A further sub-division has been made each year into full-timers and short-timers. The latter only number 167, and their heterogeneous character is fully detailed. To be included in the full-time group the length of stay of a patient at the sanatorium has been taken to be two months for the years 1905 to 1907, and as one month for subsequent years. The reason for this apparently arbitrary proceeding is that in the early years it was customary for patients to remain much longer at rest, and it was, therefore, often not until the second month that it was possible to ascertain whether a patient were febrile or not when tested by exercise. It should be remembered that the great majority of the cases sent to Frimley were afebrile on admission.

From the above account of the sub-divisions it will be seen that from the thirty-six tables a great deal of interesting, not to say important, information may be culled. The first series consists of the twenty-four tables relating to the after-history of the patients discharged in the six years 1905 to 1910, each table being repeated four times (for the full and short-timers and T.B. + or −). In the second series of tables the after-histories of the full-timers are further analysed according to the patient's condition on discharge. Five classes are given, of which the first, "Disease apparently arrested," deserves a detailed mention. The qualifications for this class form a useful standard which might well be followed by tuberculosis workers in future statistical compilations. They are:

(*a*) Loss of symptoms.

(*b*) Disappearance of cough and sputum; or if still slight sputum, then no T.B.

(*c*) Restoration of general health and bodily condition.

(*d*) Weight equal to or above normal standard.

(*e*) Fitness for and ability to perform work.

### Third Series of Full-time Cases.

The third series of tables shows the after-histories of all the full-time cases, both those with T.B. and those without, which could be traced for five and four years subsequent to discharge, thus providing statistics based on larger figures. Untraced cases are eliminated in working out the percentage of results obtained.

It is, of course, impossible here to give any useful *résumé* of all these tables, which are well worth a careful study, but a few samples may be with advantage taken from the third series. We find that of 292 full-time T.B. + cases discharged during 1905-6-7, 38.8 per cent. were "well and able to work" at the end of the fifth year following their discharges. The corresponding figure for 124 non-T.B. cases is 74.3 per cent.; in each case those lost sight of have been eliminated. Taking the two classes together, the percentage in the "well" group amounts to 48.4 at the end of the fifth year. Of cases discharged between 1905 and

1908, all full-timers, we find that at the end of the fourth year after discharge 44.5 per cent. of 475 cases with T.B. + and 82.9 per cent. of 215 cases T.B. – are in the first group, or together (690 cases) 55.9 per cent. were "well."

These results do not compare unfavourably with those of German sanatoria, but in view of the careful selection of cases for Frimley it might have been expected that a larger proportion would have retained their health. The difference in the figures between those with tubercle bacilli and those without is very striking, and shows the necessity of keeping these two groups separate.

As is pointed out by the authors of this report, the results are greatly influenced by the environment of the patient after he has left the sanatorium and the possibility of obtaining satisfactory work. There can be no doubt that to ensure the maximum benefit from sanatorium treatment a very much more efficient supervision must be exercised over discharged patients than is at present undertaken. The above report has been issued by the visiting physicians, Drs. Habershon, Wethered, Hartley, and Perkins, who have had the able assistance of Dr. W. O. Meek, the medical superintendent, in compiling the tables. It is published at 2s. 6d.

